# Nonspecif ic response of Lake Baikal phytoplankton to anthropogenic impact

**DOI:** 10.18699/VJGB-22-57

**Published:** 2022-08

**Authors:** A.A. Nikonova, S.S. Vorobyeva

**Affiliations:** Limnological Institute of the Siberian Branch of the Russian Academy of Sciences, Irkutsk, Russia; Limnological Institute of the Siberian Branch of the Russian Academy of Sciences, Irkutsk, Russia

**Keywords:** Baikal, phytoplankton oxidation stress, stress response in diatoms, alkylbenzene sulfonates, TBARs, adaptation of phytoplankton, Байкал, окислительный стресс фитопланктона, стресс диатомовых, алкилбензолсульфонаты, тиобарбитуровая кислота, адаптационная реакция фитопланктона

## Abstract

In this study, we present the first results on oxidation stress in Lake Baikal phytoplankton and its adaptation to environmental changes under anthropogenic impact. As was shown, the changing of the dominant species of phytoplankton collected from the surface water layer (~0.3 m) took place from February to June 2021. Phytoplankton were collected at a nearshore station (a littoral station at a distance of ~0.01 km from the shoreline, depth to bottom is ~5 m) and an offshore station (a pelagic station at a distance of ~1 km from the shoreline, depth to bottom is ~543 m). In February, dinoflagellates were dominant (~40 %) as well as diatoms (≤33 %) and green algae (≤12 %). Their biomass was 100 mg·m–3. In March, chrysophytes were dominant (up to 50 %) as well as cryptophytes (≤43 %) and dinoflagellates (≤30 %). Their biomass was 160–270 mg·m–3. In April, biomass increased up to 700–3100 mg·m–3 with the dominance of large cell dinoflagellates (up to 99 %), chrysophytes (up to 50 %), and cryptophytes (up to 35 %). By the end of the first decade of May, the percentage of dinoflagellates decreased and that of cryptophytes increased. In the second decade of May, the percentage of diatoms increased up to ~26–38 % but phytoplankton biomass was minimal (13–30 mg·m–3). By June, the percentage of diatoms in the samples reached 44–75 % at 60–550 mg·m–3. The oxidation stress of phytoplankton as a nonspecific adaptive response to a prolonged, intensive, or recurrent effect of a stress factor was estimated from the content of thiobarbituric acid reactive substances (TBARS). The mean content of these substances (markers of the lipid peroxidation) was determined spectrophotometrically. The oxidation stress of phytoplankton was revealed only when diatom algae dominated. It can be explained by adaptation of algae of other classes to the stress factor. The content of the lipid peroxidation markers in the coastal phytoplankton collected close to the settlement of Listvyanka known as a large touristic center was estimated from 100 to 500 μg·g–1 of dry weight of sample. During the period of diatom blooming in 2016 and 2018, oxidation stress of phytoplankton collected near large settlements was found. In phytoplankton from deep-water pelagic stations most remote from settlements, stress was not revealed. Using the method of gas chromatography, we showed a lower (up to 15 %) content of polyunsaturated fatty acids in phytoplankton characterized by stress occurrence. This confirms cell membrane damages. In Lake Baikal surface water, we found a higher content of synthetic anionic surfactants (sodium alkylbenzene sulfonates), which are components of detergents and cause oxidation stress of hydrobionts (up to 30 ± 4 μg·L–1). The presence of these substances in a water ecosystem can result in exhausting of phytoplankton cell resources, homeostasis imbalance, stress, pathological changes, and rearrangements in phytoplankton assemblage

## Abstract (ru)

Представлены первые результаты по изучению окислительного стресса фитопланктона из озера Байкал и его адаптивных свойств к изменению среды обитания в условиях повышенной антропогенной нагрузки. Анализ фитопланктона, отобранного в поверхностном слое воды (~0.3 м) на прибрежной (глубина 5 м, расстояние от берега 10 м) и пелагической станциях (глубина 543 м, расстояние от берега 1000 м), показал смену доминирующих видов с февраля по июнь 2021 г. В феврале доминировали динофитовые (~40 %), диатомовые (до 33 %) и зеленые (до 12 %) водоросли с низкой биомассой – 100 мг/м3. В марте преобладали золотистые (до 50 %), криптофитовые (до 40 %) и динофитовые (до 30 %) (биомасса 160–270 мг/м3). В апреле наблюдалось увеличение биомассы до 700–3100 мг/м3 с доминированием крупноклеточных динофитовых (до 99 %), золотистых (до 50 %) и криптофитовых (до 35 %) водорослей. К концу первой декады мая доля динофитовых снизилась и увеличилась доля золотистых. Уровень развития диатомовых повышался во второй декаде мая до ~26–38 % при минимальной биомассе фитопланктона (13–30 мг/м3). К июню доля диатомовых в пробах достигала 44–75 % при биомассе 60–550 мг/м3. Окислительный стресс фитопланктона как неспецифическую адаптационную реакцию на длительное, интенсивное либо повторяющееся воздействие стресс-фактора оценивали по содержанию в пробах веществ, вступающих в реакцию с тиобарбитуровой кислотой. Среднее содержание данных веществ – маркеров перекисного окисления липидов – было оценено спектрофотометрически. Окислительный стресс фитопланкто- на выявлен только в период доминирования в пробах диатомовых водорослей. Это может быть объяснено луч- шей адаптацией водорослей других отделов к стресс-фактору. Содержание маркеров перекисного окисления ли- пидов в прибрежном фитопланктоне, отобранном вблизи населенного пункта и крупного туристического центра пос. Листвянка, составило от 100 до 500 мкг/г сухой массы пробы. В 2016 и 2018 гг. в период массового развития диатомовых водорослей обнаружен окислительный стресс фитопланктона вблизи крупных населенных пунктов. В фитопланктоне глубоководных пелагических станций, максимально удаленных от населенных пунктов, стресс не найден. С помощью метода газовой хроматографии показано более низкое (до 15 %) содержание полиненасы- щенных жирных кислот в планктоне, характеризующемся наличием стресса. Это свидетельствует о повреждении мембран клеток. Повышенное содержание анионных синтетических поверхностно-активных веществ, а именно алкилбензолсульфонатов натрия, являющихся компонентами моющих средств и вызывающих окислительный стресс гидробионтов, обнаружено в поверхностной воде оз. Байкал (до 30 ± 4 мкг/ дм3). Наличие данных соеди- нений в водной экосистеме может приводить к исчерпанию ресурсов фитопланктона, нарушению гомеостаза, стрессу, патологическим изменениям и перестройкам планктонного сообщества.

## Introduction

Under the effect of external factors on a living cell and the organism as a whole, a complex of nonspecific and specific adaptive defense responses occurs. Nonspecific responses also called stress are the response of a living system to intense or unusual irritants. This allows assessing the scale of the impact of stress factors on an organism. In this case, adaptation mechanism includes the activation of all systems of an organism counteracting stress factors and supporting homeostasis and dynamic balance between the organism and the environment. Rate of exposure to chemicals as a cell stress factor varies depending on their characteristics (concentration, physical and chemical properties of the molecules) as well as on the individual response and the adaptation potential. In the case of prolonged, repeated, or intense exposure, a malfunction of the organism’s adaptive reactions may occur. This leads to resource depletion, homeostasis imbalance, distress, and pathologies (Poryadin, 2009).

Pollution of aquatic ecosystems by xenobiotics and absence of adaptation of water dwellers to their effect are an acute problem for the 21st century. Some of the most common persistent micropollutants in aquatic ecosystems are polycyclic aromatic hydrocarbons (PAH) (Vega-López et al., 2013) and heavy metals (Srivastava et al., 2006). Alkylbenzene sulfonates are the most common persistent macropollutants (Lewis, 1991; Jorgensen, Christoffersen, 2000). The common feature of the substances mentioned above is their ability to induce oxidative stress and hypoxia of the cell and organism as a whole

The increasing activity of the enzymes such as superoxide dismutase, catalase, and glutathione peroxidase due to initial or minor stress is an indicator of the oxidation stress, which is a nonspecific adaptive response. On the contrary, prolonged or intense exposure to a stress factor may result in suppressing of the effects of enzyme activity of a living organism, disease, and death. The aldehydes including malone dialdehyde being formed due to the destruction of the lipids of the cell membranes by reactive oxygen species (lipid peroxidation) is another indicator of oxidation stress (Marnett et al., 1999; Hampel et al., 2008; Goncalves et al., 2017; Zhou et al., 2018; Nikonova et al., 2022).

Aquatic microorganisms such as phytoplankton are capable of activating the defense systems of the organism such as the hormonal system, adenine nucleotide exchange system, prostaglandin and antioxidant systems. The latter is better investigated. It usually allows resistance to natural physical and chemical factor effects but can not cope with xenobiotic impact (Karthikeyan et al., 2013). Phytoplankton are extremely sensitive to environmental changes. The state of the entire ecosystem depends on their well-being.

Particular attention should be paid to diatoms, which are good indicators of water quality. Diatoms are used in the biomonitoring of heavy metals and organic pollutants such as petroleum, polyaromatic hydrocarbons (PAH), pesticides, polychlorinated biphenyls (PCB), and anionic surfactants (Datta et al., 2019). This is due to diatoms being considered the most diverse phytoplanktonic group in all aquatic ecosystems. Many of them are common to water bodies of different types and live all over the world. This allows comparing the data of the analysis. Rapid diatom response to both short-term and long-term physical and chemical environmental changes is noticed (Dixit et al., 1992). Different sensitivity of various species of diatoms is known (Datta et al., 2019). For example, some of them are susceptible to eutrophication (Eunotia sp., Diatoma vulgare, Gomphoneis herculeana, Achnanthidium sp., Achnanthes subhudsonis var. kraeuselii), effects of organic pollutants (Nitzschia palea, Nitzschia fonticola), heavy metals (Fragilaria capucina, Achnanthidium minutissimum), electroconductivity fluctuations (Fragilaria ulna var. acus (Kütz.) Lange-Bert.), pH changes (Eunotia sp., Pinnularia sp., Eunotia exigua, Gomphonema angustum, Amphora veneta, Gomphonema rautenbachiae), flow rate (Melosira sp., Cocconeis sp.), mass transport and sedimentation (Navicula sp., Nitzschia sp., Surirella sp.), concentration of nutrients such as nitrogen (Gomphonema parvulum, Eolimna minima, Nitzschia palea) and phosphorous (Gomphoneis herculeana, Achnanthidium sp., Achnanthes subhudsonis var. kraeuselii, Luticola goeppertiana, Navicula recens, Nitzschia inconspicua, Nitzschia palea, Rhopalodia sp., Eunotia sp.), and others.

Lake Baikal is the deepest and oldest rift lake containing 23,615.39 km3 of ultra fresh water with a total mineralization of 96–98 mg · L−1. Due to basin peculiarities, the surface area of Lake Baikal is 32,822 km2, of which the littoral zone occupies only ~3.4 %. The littoral contains maximal biodiversity (more than 98 % of species) with biomass up to ~100–620 kg per hectare at depths of <4–70 m. Phytoplankton inhabit the littoral and the pelagic zones down to ~750 m of depth but its maximal abundance characterizes the photic zone at depths of ~60–120 m and the zone of the intensive vertical water mixing by wind at depths up to ~200–300 m. In the spring season, phytoplankton primary production reaches ~160 tones per hectare (Votintsev et al., 1975; Nikonova et al., 2022). About 200 species of planktonic algae were registered in the water column of the lake. More than 50 of them are diatom species (Votintsev et al., 1975; Rusinek, 2012). The percentage of diatoms reaches 50–90 % of the total phytoplankton biomass (Popovskaya et al., 2015). The littoral is much more exposed to negative anthropogenic impact than the pelagic zone. Since 2000, changes in nearshore phytoplankton have already been observed (Bondarenko, Logacheva, 2017). In 2019, the oxidation stress of the coastal phytoplankton was described (Nikonova et al., 2022) but the reasons behind this phenomenon have not yet been established unambiguously.

The objectives of our study were to evaluate the nonspecific adaptive response of Lake Baikal phytoplankton to anthropogenic impact and to assess the possibility of using them as a bioindicator.

## Materials and methods

Water sampling for determining the phytoplankton composition. All samples were collected from stations of three types: nearshore stations (depth to bottom up to 30 m); short-distance pelagic station (distance from the shoreline ~1–3 km); central pelagic stations (distance ~10–30 km both from the east and west shorelines).

Sampling was carried out in 2021 during the under-the-ice period from the third decade of February to the first decade of May and during the open water period from the third decade of May to the first decade of June. Water from the surface down to a depth of 0.5 m was sampled regularly at the stationary stations to analyze the phytoplankton composition. The nearshore stationary station is characterized by the depth to bottom ~5 m and the minimal distance from the shoreline of 10 m. The short-distance pelagic station is characterized by the depth 543 m and the distance from the shoreline of 1000 m. The stationary stations are situated opposite the Sennaya River mouth in Listvennichnyi Bay located in the southern basin of the lake. Besides, phytoplankton were sampled in three basins of Lake Baikal in 2016 and 2018. Water samples of 1 L volume were poured into bottles and fixed with Lugol’s solution. Then, samples underwent concentration according to the classical method by cell sedimentation during 10 days at room temperature in the dark (Nakashizuka, Stork, 2002). The concentrates were used to assess species composition, number of cells, and biomass.

Net sample collection. The representative samples of phytoplankton biomass were obtained using the Juday-type net with 100 μm mesh size. Live phytoplankton collected at the stationary sampling sites were transported to the laboratory in thermoses and filtered through the cellulose acetate filters (0.45 μm, Vladisart, Russia) using the filter-apparatus (Duran Group, Germany). The lipid peroxidation markers were analyzed without delay. The residual biomass was wrapped in aluminum foil and stored at −70 °C. The samples of phytoplankton biomass, which could not be transported to the laboratory as live biomass, were filtered, frozen at −20 °C, transported to the laboratory, and stored at −70 °C.

Microscopy and estimation of phytoplankton qualitative characteristics. Cells in concentrated sedimentation samples were subsequently identified using a light microscope Amplival (Carl Zeiss, Jena) at ×800 magnification. Species diversity was estimated according to conceptual guides for measuring species diversity (Matvienko, Litvienko, 1977; Starmach, 1985; Round et al., 1990; Tsarenko, 1990; Glezer et al., 2011). Cell number in each sample of 0.1 mL volume (N, cells·103/mL) was counted according to formula where N is average cell number per volume, V1 – volume of decanted water, V2 – volume of the concentrated sample.

**formula:**
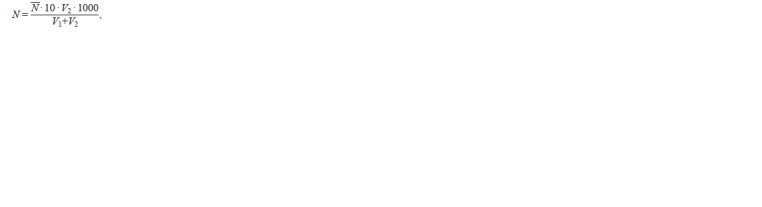
formula

The cell number was converted into the cell biomass (B) taking into account the individual shape, size, and volume of the cell of every species (Makarova, Pichkily, 1970; Belykh et al., 2011).

Water sampling for determining the concentration of anionic surfactants. The surface water of 0.1 L volume was collected from Lake Baikal at depths up to 0.5 m from 30 May to 18 June 2021 using the bathometer to analyze anionic surfactant concentrations. Water samples were placed into dark glass bottles and fixed with ethyl alcohol (1 mL). To analyze anionic surfactant and phytoplankton composition both samples were collected at the same time from 30 March to 18 April 2021. Water was also sampled in the mouths of rivers Sennaya, Bannyui Ruchei, Krestovka, Bolshaya Cheremshanka, and Malaya Cheremshanka, inflowing into Lake Baikal. Samples were filtered through micro-cellulose acetate filters (0.45 μm, Vladisart, Russia) using the filter-apparatus. After that, the filter with suspended matter was cut and put into the 10 mL glass flask. To extract the anionic surfactants, 1 mL of distilled ethyl alcohol was added to each sample. Then, samples underwent extraction for 5 min using a 50 Hz ultrasonic bath. After that, the extracts were placed in plastic 2-mL Eppendorf taste-tubes and centrifuged at 13,000 rpm for 3 min. The supernatant was merged with the filtered water, and the obtained samples were stored at +3 °С until the analysis.

Identification of alkylbenzene sulfonates in water samples. The identification of alkylbenzene sulfonate homologues in water sample extracts concentrated onto DSC-18 reversed-phase sorbent (0.5 g, Supelco, USA) was carried out with a reversed-phase high performance liquid chromatograph Milichrom А-02 (Eco-Nova, Russia) coupled to a UV-detector. A water solution of linear alkylbenzene sulphonate (LAS) mixture (GSO 8578-2004) was used as an external standard (100 mg/mL, Analytic-Chim, Russia). Chromatography was performed at 60 °С using 2 × 75 mm Nucleosil 100-5-С18 column (Eco-Nova, Russia). The characteristics were the following: solvent A – water with 0.1 % (v/v) trifluoroacetic acid (TFA); solvent B – acetonitrile (ACN) with 0.1 % (v/v) TFA; isocratic – 40 % B in 0.3 mL; then gradient – 40–100 % B in 2 mL; injection volume – 100 μL; detection – UV 224 and 230 nm

The determination of anionic surfactants. Spectrophotometric qualitative analysis of anionic surfactants in water samples collected in Lake Baikal and its tributaries was implemented using methylene blue. The samples of 50 mL volume were extracted with chlorophorm according to previous work (Nikonova et al., 2022). A double beam UV-Vis Cintra-20 spectrophotometer (GBC, Australia) with a Czerny–Turner configuration monochromator and holographic diffraction grating provided precision and accuracy of the obtained data. Standard quartz cuvettes of 1 cm path length were used. Absorbance was measured at 651.5 nm.

Qualitative and quantitative analyses of fatty acids. To extract fatty acids (FA), 1.2 mL of Folch solution (chloroform– methanol, 2:1 by volume) was added to each sample, then it was placed into an ultrasonic bath (3 × 5 min) (Nikonova et al., 2020, 2022). After that, 0.35 mL of distilled water was added to the extracts (chloroform–methanol–water partition 2:1:1 v/v). The mixtures were vigorously shaken and centrifuged at 3,000 rpm for 3 min. The supernatant was put into glass vials and the solvent was evaporated using an argon stream. Then, 4.5 mL of 2 % H2SO4 solution in methanol was added to the dry fraction. Esterification of fatty acids was carried out at 55 °C during 1.5 h. Fatty acid methyl esters (FAMEs) were extracted with n-hexane (3 mL × 2 × 2 min). The extracts were dried with anhydrous Na2SO4. The internal standard (1 mg·mL–1 of do-decyl ether solution in n-hexane) was added to the extract. The sum analysis of both free and etherified FAs by gas chromatography coupled to mass-spectrometry as well as by gas chromatography coupled to flame ionization was carried out using the “6890В GC System, 7000С GC/MS Triple Quad” (Agilent, USA) and “GC-2010 Plus” (Shimadzu, Japan) with “Optima-17MS” 30 m × 0.25 mm columns (Macherey-Nagel, Germany).

Estimating the oxidation stress of phytoplankton. To estimate the oxidation stress of phytoplankton, we analyzed thiobarbituric acid (TBA) reactive substances (TBARS). To prepare the samples of 0.15–0.20 g of weight, we used the analytical method described earlier (Haraguchi et al., 1997; Al-Rashed et al., 2016) with our modifications (Nikonova et al., 2022). The analysis was carried out with a Cintra-20 spectrophotometer

## Results

Twenty genera of microalgae including 39 taxa of phytoplanktonic algae and 21 taxa of benthic algae collected both at the nearshore station (depth to bottom ~5 m, distance from the shoreline 10 m) and at the pelagic one (depth to bottom 543 m, distance from the shoreline 1000 m) were identified from February 23 to May 26 in 2021. Among phytoplanktonic algae, 7 classes were identified: chrysophytes (5 taxa), blue-green algae (3 taxa), cryptophytes (4 taxa), dinoflagellates (7 taxa), diatoms (11 taxa), green algae (8 taxa), and euglenophytes (1 taxon). The total number of the samples was 23.

In the nearshore zone, phytoplanktonic algae biomass varied significantly from 13.4 to 3111 mg · m–3, and the dominant species of phytoplankton changed in the period mentioned above. Dinoflagellates Gymnodinium baicalense and Peridinium baicalense (~40 %), diatoms Synedra acus subsp. radians (up to 33 %), and green algae Monoraphidium arcuatum (up to 12 %) were dominant in February. Their total biomass (102 mg · m–3) and cell number (100 · 103 cells · L–1) were small. Changes in phytoplankton composition with the dominance of chrysophytes Dinobryon cylindricum (25–50 %), cryptophytes Rhodomonas pusilla (15–36 %), and dinoflagellates (~30 %) were fixed in March. Their biomass reached 160–270 mg · m–3 and their cell number was (130–170) · 103 cells · L–1.

The maximal phytoplankton biomass (3110 mg·m–3) and the maximal cell number (1030 · 103 cells·L–1) were registered in April. Dinoflagellates G. baicalense and P. baicalense (92– 99 %) dominated till the third decade of April. The maximal biomass was recorded before the end of the first decade of May (930 mg·m–3) and was defined by chrysophytes (~63 %), cryptophytes (~18 %), and diatoms (~16 %).

By the end of the third decade of May, the maximal biomass decreased down to minimal values (13–30 mg·m–3). A decrease in phytoplankton growth and changes in the phytoplankton assemblage structure were fixed. Diatoms (30–40 %) and cryptophytes (20–30 %) were the dominants in contrast to chrysophytes (13–20 %), dinoflagellates (~15 %), and green algae (6–16 %).

In the pelagic zone, the qualitative characteristics of the phytoplankton were low at the end of March (N = 35 · 103 cells·L–1, B = 93 mg·m–3). Dinoflagellates (up to 50 %), cryptophytes (up to 30 %), and chrysophytes (up to 20 %) prevailed. By the end of the first decade of April, dinoflagellates G. baicalense and P. baicalense (up to 90 %) dominated (N = 100 · 103 cells·L–1, B = 900 mg · m–3). In the middle of April, the dominants were the same but the cell number reached 200·103 cells·L–1 and the biomass increased up to 1600 mg · m–3.

In the first decade of June, diatoms dominated at the stationary stations (up to 80 %). The detailed composition of Lake Baikal phytoplankton collected in Listvennichnyi Bay during the diatom bloom period is given in Table 1.

**Table 1.**
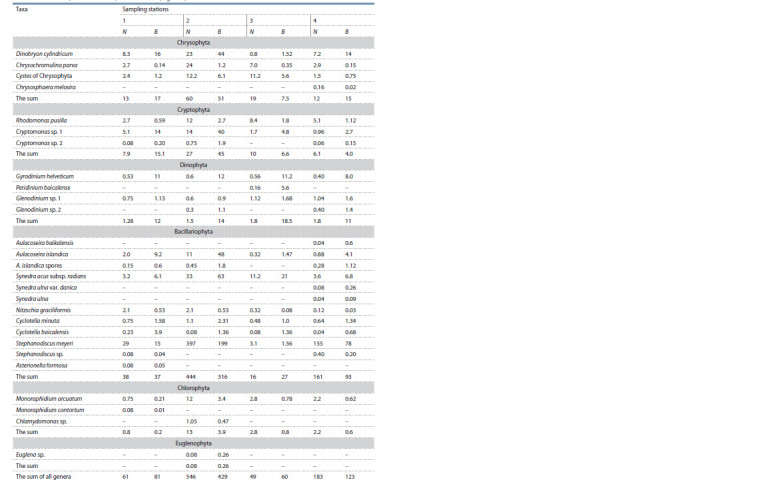
Composition of Baikal phytoplankton sampled in Listvennichnyi Bay (June 5, 2021) and its quantitative characteristics such as cell number (N · 103 cells · L–1) and biomass B (mg · m–3) The coastal stations are marked by Nos. 1–3 and the pelagic one is marked by No. 4.

**Fig. 1.**
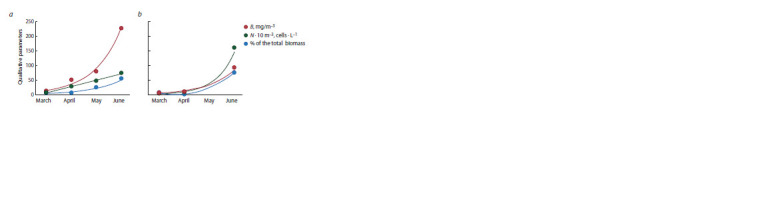
The increase in quantitative characteristics of phytoplankton in 2021: a, nearshore phytoplankton; b, pelagic phytoplankton.

The increase in quantitative characteristics of phytoplankton is shown in Figure 1. These are the percentages of the total biomass, cell number, and the biomass of diatoms of Lake Baikal phytoplankton collected in Listvennichnyi Bay during the spring season of 2021.

We did not find lipid peroxidation products (LPOP) in net samples of phytoplankton collected in March–April before the intense diatom bloom. During the period of intense diatom blooming LPOP were not revealed in four pelagic samples, and in two other samples their contents were minimal (13 and 50 μg·g–1 of dry weight (d. w.)). LPOP content in nearshore phytoplankton was estimated in a range from 120 to 630 μg · g–1 d. w. The samples of nearshore phytoplankton were collected at two sample sites: (1) the stationary station in front of the River Sennaya and (2) the station in front of the settlement of Listvyanka. Two independent net samples were collected at each site. Three sub-samples were picked from each sample excluding a step of biomass homogenization to estimate the distributional heterogeneity of the analyzed substances. The increase in the level of phytoplankton oxidation stress was fixed at the sample site No. 1. The content of LPOP reached 120–240 μg · g–1 in biomass collected from the site No. 1 and in biomass collected from the site No. 2 it was 540–630 μg · g–1 (Table 2).

**Table 2.**
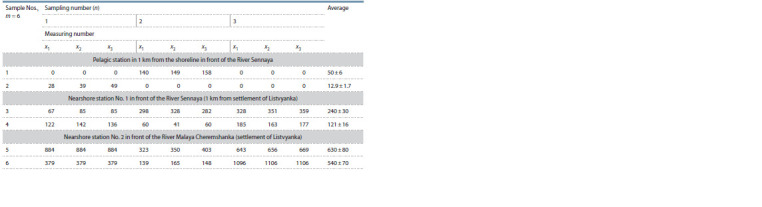
LPOP content (μg·g–1 of dry weight) in phytoplankton sampled from the stationary stations

**Fig. 2.**
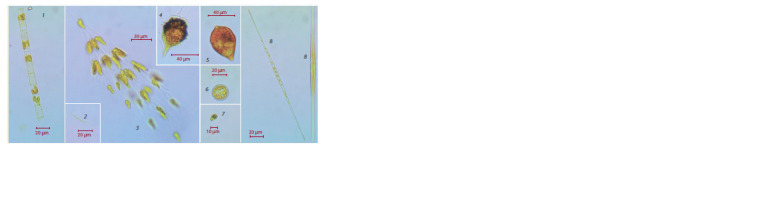
Spring phytoplankton of Lake Baikal collected in Listvennichnyi Bay: 1, A. islandica, 2, Kolliella longista, 3, D. сylindricum, 4, Peridinium baicalense, 5, G. helveticum, 6, C. minuta, 7, Rh. pusilla, 8, S. acus subsp. radians. The photos were obtained with the use of a LOMO Micromed-6 light microscope at ×400 magnification

The diatom S. acus subsp. radians prevailed (92–95 %) in phytoplankton samples (m = 20) collected in three basins of Lake Baikal during the first decade of June in 2016, 2018 (Fig. 2). Diatoms of other species as well as chrysophytes contribute ≤5 % to the total biomass. This allowed us to compare the characteristics of the samples taken from different sites. The pelagic sample stations were located in the center of the lake (m = 3). Among the nearshore stations, background stations (m = 4) as well as the sites located near the cities and large settlements (m = 9) were chosen. The samples of the axenic laboratory culture of S. acus subsp. radians were also analyzed (m = 3) (Table 3). In nearshore phytoplankton, LPOP content as a marker of oxidation stress was estimated in a range from 14 to 340 μg · g–1 d. w. and it was not found in the biomass of pelagic phytoplankton collected from the central stations.

**Table 3.**
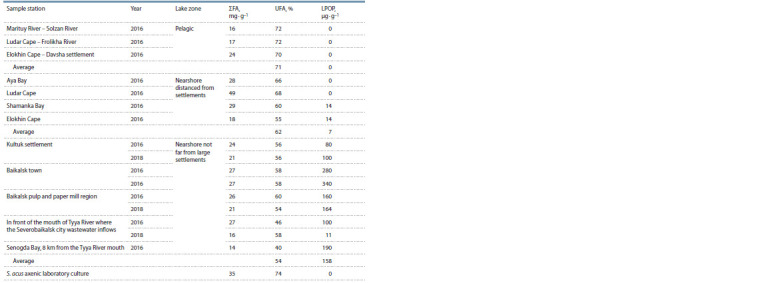
Contents of the unsaturated fatty acids and oxidation stress marker substances in phytoplankton with the diatoms as a dominant The limit of LPOP determination (LOD) of 0.5 μmol·mL−1 via spectrophotometry was determined by Rakita et al. (2020); UFA – unsaturated fatty acids, which means the sum of all monounsaturated and polyunsaturated fatty acids.

**Fig. 3.**
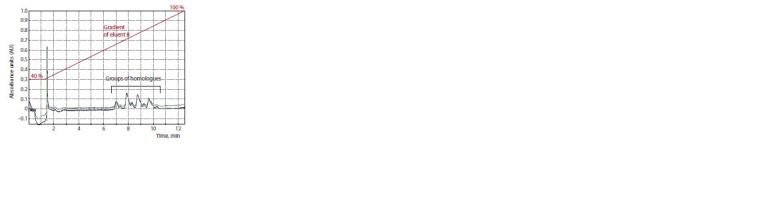
The chromatogram of alkylbenzene sulfonates homologues in water from Listvennichnyi Bay (April, 2022). Peaks identification was carried out according to FR.1.38.2017.27043 method of sodium alkylbenzene sulfonate (sulfanol) determination by HPLC-UV (in Russian).

Anionic surfactants were found in all of the water samples. The qualitative composition was represented by homologues of sodium alkylbenzenesulfonate (Fig. 3). The concentration of these pollutants in water of the nearshore zone close to large settlements achieved 21 ± 3 μg·L–1. It was less than 10 μg ·L–1 in water of the background stations and less than 5 μg ·L–1 in pelagic water. Anionic surfactants concentrations in water of Lake Baikal tributaries such as the Rivers Bolshaya Cheremshanka (12.6 ± 1.5 μg ·L–1), Malaya Cheremshanka (8.1 ± 1.0), Krestovka (74.5 ± 9.0), Bannyi Ruchei (14.8 ± 1.8), Sennaya (30.1 ± 3.7) were found in a wide range. The Krestovka River flows through the Listvyanka settlement and is characterized by maximal discharge and surfactants concentrations in water.

## Discussion

For Lake Baikal, marine peculiarities of climate as well as a delay of seasonal onsets in the coastal zone are common compared to the nearby continental zone (Ladeishchikov, 1987). That is why June is a spring month at the Lake Baikal territory. Analysis of phytoplankton collected at stationary sites during the spring season (March–June 2021) shows clear changes in dominant species. Diatom abundance increased from 5 % in March–April to 44–75 % in the first decade of June. This event has been noticed earlier and is typical for Baikal (Vorobyeva, 2018).

High contents of monounsaturated (MUFA) and polyunsaturated fatty acids (PUFA) characterize the planktonic assemblage with the Synedra acus subsp. radians as a dominant. The major fatty acids (FAs) are С18:3-ω-3 (~7–9 %) and С20:5-ω-3 (~10–23 %, average 17 %). С20:4-ω-6 and С22:6-ω-3 FAs are presented in less content (Nikonova et al., 2020). The mentioned FAs are the most destructible under the free radical effect. Polyunsaturated FAs are mostly concentrated in lipid bilayer of the cell membranes. The membrane of S. acus is covered with a silica cell wall and contains ~30 % of PUFA, which makes it vulnerable to free radical attack.

**Fig. 4.**
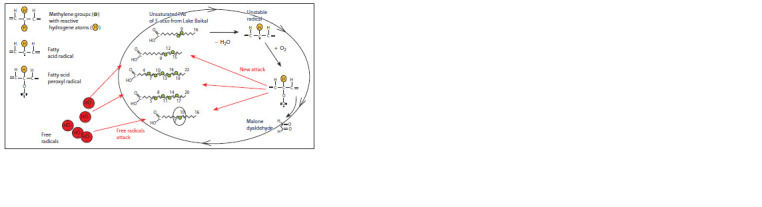
Free radical mechanism of preliminary unsaturated FAs peroxydation of Lake Baikal phytoplankton with the S. acus dominance.

There are two known routes for lipid destruction in the cell. The first is the α-, β-, and ω-oxidation of lipids by enzymes with the formation of numerous vital compounds. The second is the lipid peroxidation. The final products of peroxidation are peroxides and aldehydes including malondialdehyde (MDA). Peroxidation of unsaturated FAs takes place in the case of free radical attack of reactive hydrogen atoms of the methylene group of the alkyl chain. These groups should be conjugated to a pair of the C–C double bonds. The lipid peroxyl radical, which formed as a result of the mentioned process, then reacts with another fatty acid to produce a new lipid radical and lipid hydroperoxide; thus, this chain reaction continues (Fig. 4).

Nonspecific adaptation response of the cell and the organism as a whole is the response to a stress factor effect, which is common for different organisms. This response aims to restore the homeostasis of the system. An example of a nonspecific response is oxidation stress. For instance, the oxidation stress of green algae due to UV-B (Al-Rashed et al., 2016) and the oxidation stress of aquatic plants as a result of heavy metals impact (Srivastava et al., 2006) were described. The oxidation stress of Lake Baikal phytoplankton during the intense diatom bloom found by us is an unspecific adaptation response to environmental changes. Nevertheless, the data of the analysis of phytoplankton with chrysophytes, cryptophytes, dinoflagellates as dominant species show the absence of oxidation stress markers. This is related to cell membrane structure of the mentioned algae, which contains cellulose and hemicellulose. It makes the membrane more resistant to free radical impact and enables a better adaptation of these microalgae.

The significant inhomogeneity of the results of LPOP determination was noticed when collecting two samples at each station. The relative standard deviation reached 90 %. Because of the high reactivity of free MDA, the results of determination of this substance content in the biological samples are usually understated. This marks the lipid peroxidation process, which is taking place in a cell at the moment (Zelzer et al., 2013). Inhomogeneity of the results is most probably induced by the high reactivity of MDA. So, the LPOP determination results evidently can not provide the precise qualitative characteristic of the stress. Though the LPOP occurrence unambiguously confirms the diatom cell membrane potential exhaustion, malfunction of the adaptive reaction, homeostatic imbalance, and the evident oxidation stress of the phytoplankton collected in the regions of a high intensity anthropogenic load.

The absence of the oxidation stress of the pelagic phytoplankton from the central stations, as well as lower oxidation stress of the phytoplankton collected from the background nearshore stations and the UFA content decrease in stressed phytoplankton confirm the correlation of the stress of S. acus with the effects of a stress factor (see Table 3). The last one is unusual for the species mentioned above, and the protective adaptation mechanism has not formed yet.

The authors of this work suggest sodium linear alkylbenzene sulfonates to be a potential stress factor resulting in the lipid peroxidation of Baikal diatoms. The concentrations of these pollutans in surface water achieved critical values up to 30 ± 4 μg · L–1 near large settlements and cities in 2019–2021 though. In the only sample concentration reached 54 ± 7 μg · L–1 though for the most of samples it does not exceed 10 ± 1.2 μg · L–1. Surfactants of this type possess maximal hazard, and their affect causes acute toxicity to water organisms, as well as chronic influence including oxidation stress at ≤10–20 μg · L–1 (Lewis, 1991; Jorgensen, Christoffersen, 2000). Anionic surfactants and alkylbenzene sulfonates in particular were related to hazard substances1 according to the United Nations Environment Programme (UNEP) and to especially hazard substances2 for the unique ecosystem of Lake Baikal presented as a UNESCO World Heritage Site.

1 Linear alkylbenzene sulfonates. SIDS Initial Assessment Report for 20th SIAM. UNEP Publications, Paris, France, 19–21 April, 2005. 2 Order of Russian Federation No. 83 (21.02.2020). On approval of standards of maximum permissible actions on unique ecological system of Lake Baikal and list of hazardous substances including most dangerous substances, high dangerous substances and moderate dangerous substances for unique ecological system of Lake Baikal. The Ministry of Natural Resources and Ecology of Russian Federation. 

## Conclusion

The oxidation stress of nearshore Baikal phytoplankton with diatoms Synedra acus subsp. radians as a dominant was revealed in regions of increased anthropogenic load. An assumption that S. acus is a susceptible bioindicator to xenobiotic effect causing the oxidation stress is proposed. During the under-the-ice period, the oxidation stress of phytoplankton was not found, which can be explained by the domination of the algae of other classes and their better adaptation to reactive oxygen species effect. We believe the nearshore phytoplankton stress to be caused by local critical concentrations of anionic surfactants in the coastal water of Lake Baikal.

## Conflict of interest

The authors declare no conflict of interest.
